# Molecular Epidemiology of Methicillin-Resistant *Staphylococcus aureus* Clinical Isolates during 7.5 Years in One Regional Hospital in Israel

**DOI:** 10.1155/2021/6643108

**Published:** 2021-03-05

**Authors:** Regev Cohen, Svetlana Paikin, Talya Finn, Frida Babushkin, Einav Anuka, Moti Baum, Assaf Rokney

**Affiliations:** ^1^Head of Infectious Diseases Unit and Infection Control Unit, Sanz Medical Center, Laniado Hospital, 16 Divrei Haim St. Kiryat Sanz, 42150 Netanya, Israel; ^2^The Ruth and Bruce Rappaport Faculty of Medicine, Technion, Haifa, Israel; ^3^Microbiology Laboratory, Sanz Medical Center, Laniado Hospital, Netanya, Israel; ^4^Infectious Diseases Unit and Infection Control Unit, Sanz Medical Center, Laniado Hospital, Netanya, Israel; ^5^Central Laboratories, Ministry of Health, Jerusalem, Israel

## Abstract

**Background:**

The clonal repertoire of community-associated Methicillin-resistant *Staphylococcus aureus* (CA-MRSA) strains appear to differ between hospitals and geographic locations. We aimed to study the molecular epidemiology of MRSA infections in our regional hospital in Israel.

**Methods:**

A retrospective analysis of MRSA isolates from hospitalized patients, which underwent *spa* typing between 2012 and 2019. Mainly, MRSA-bloodstream isolates were typed. Isolates were grouped into healthcare-associated (HcA) or community-associated (CA). HcA were further divided into hospital-related or long-term care facility- (LTCF-) related. Several representatives underwent SCC*mec* typing.

**Results:**

We analyzed 166 clinical MRSA isolates: 115 (70%) bloodstream, 42 (25%) wounds/abscesses, and 9 (5%) screening isolates. 145 (87%) were HcA, and 21 (13%) were CA. Common (72%) *spa* types were t002, t032, t008, t001, and t065. Eighty (55%) isolates were attributed to LTCFs and 65 isolates to our hospital, both showing similar *spa* types distribution. The most prevalent *spa* type among patients with HcA infection was t002 (50 isolates, 32%), followed by t032, t065, t578, t008, and t001. Most (88/115, 77%) bloodstream infections (BSIs) were HcA, typically occurring in the same facility in which the infection was acquired. In 27 cases (23%), the BSI developed in the community setting, and in half of these cases, a previous healthcare system exposure was evident.

**Conclusions:**

The MRSA clonal population in this longitudinal study was stable and consisted mainly of molecular lineages widespread in Europe. SCC*mec*-IV strains play a major role in causing MRSA infections in the healthcare settings, especially in LTCFs. Community-acquired MRSA BSIs without any previous healthcare exposure are still relatively rare.

## 1. Introduction

Methicillin-resistant *Staphylococcus aureus* (MRSA) is one of the most important pathogens causing severe community- and healthcare-associated (HcA) infections [1]. Methicillin resistance is coded on the Staphylococcal chromosomal cassette *mec* (SCC*mec)* element. HcA-MRSA infections were traditionally associated with SCC*mec* types I, II, and III; but in the last two decades, community-associated- (CA-) MRSA infections have emerged, resulting in skin and soft tissue, as well as invasive infections, among healthy young populations with no traditional risk factors for MRSA infections. These CA-MRSA clones are associated with SCC*mec* types IV and V, which are smaller cassettes that lack resistance genes to non-beta-lactam antimicrobials. These clones have penetrated into healthcare facilities resulting in nosocomial outbreaks. With time, the epidemiological, as well as the molecular, distinctions between HcA- and CA-MRSA infections have blurred [2]. Geographical location also influences the type of MRSA strain seen, with different strains typically seen in different continents and countries [1]. In a recent review, the most prevalent *Staphylococcus aureus* protein A (*spa*) types were t032, t008, and t002 in Europe; t037 and t002 in Asia; and t008, t002, and t242 in America. Several *spa* types are strictly related to SCC*mec*-I and II (such as t001), several are strongly related to SCC*mec*-IV (such as t032 and t008), and several may be related to both types of SCC*mec* (such as t002, which may be typed as SCC*mec*-I, II, III, IV, and V) [3].

The clonal epidemiology of MRSA strains in Israel remains largely unexplored. Most of the peripheral laboratories in Israel submit clinical isolates of MRSA cultivated from wounds and bloodstream to the Ministry of Health (MOH) central laboratories. Methicillin-sensitive *Staphylococcus aureus* (MSSA) isolates cultivated from the bloodstream are also requested. The bloodstream isolates originate mainly from hospital-based laboratories. MOH laboratory annual reports from 2016 and 2017 show that the most common MRSA *spa* types in Israel are t002, t008, t032, and t991 [4, 5], resembling other European countries. Most of the t002 isolates are from the Clonal Complex 5 (CC5), bearing the SCC*mec* type II (New York/Japan clone), but several are of SCC*mec* type V. The t008 (CC8) is typically of CA-MRSA (several of them are USA300 and USA1100 strains); t032 is from the CC22 (EMRSA15), which is common in Europe, and t991 (CC913, SCC*mec*-IV) is typically found in pediatric patients in Israel [6, 7]. The *spa* types of MSSA strains in Israel are much more diverse than those of MRSA.

During 2006–2010, the Israeli National Center for Infection Control performed a hospital-based survey of MRSA from 5 hospitals across Israel. The most common MRSA strains they found belonged to common epidemic clones: t001/SCC*mec*-I, t002/SCC*mec*-II, t065/SCC*mec*-IV, t008/SCC*mec*-IV, and t051/052/SCC*mec*-I clones. SCC*mec* IV and V proportions were relatively high in this study (27%) as compared with other countries, and the study also reported on clones that were unique to Israel (such as t002/SCC*mec*-V) [8]. A more recent, hospital-based study from the center of Israel, which also included surveillance isolates, found that SCC*mec* types IV and V were common in the hospital settings. SCC*mec*-V was seen more among patients from Arab ethnicity, and there were no differences between patients with SCC*mec* types I–III and patients with SCC*mec* types IV-V [9]. Only 3% (16/501) of cases in this study had no obvious exposure to the healthcare system. This study implies that SCC*mec* types IV and V often cause healthcare-associated MRSA infections after exposure to hospitals and long-term care facilities (LTCFs), although it is important to note that screening policy in this study was focused on high-risk populations, such as LTCFs dwellers; thus, the results may be biased.

A community-based study from Israel (conducted in 2006) screened 3373 children and their parents visiting 53 primary care clinics and found 580 to be carriers of *S. aureus*, of which only 5 were MRSA. Two of the five isolates were defined as CA-MRSA based on epidemiological data, antimicrobial susceptibility, and the presence of SCC*mec*-IV [10]. On the other hand, a more recent community-based study of all clinical MRSA isolates collected during the years 2011–2013 in one major health-maintenance organization (HMO) found that 43% and 9% of all MRSA isolates were SCC*mec*-IV and V, respectively. Of the SCC*mec*-IV, *spa* types t032, t008 (USA300), and t991 were the most common. Patients with SCC*mec* type IV were younger and were less frequently hospitalized as compared with patients with types I–III [7]. A study of the pediatric population of southern Israel reported a high prevalence of MRSA nasal carriage (4.4%) among Bedouin children, mainly of SCC*mec-*IV (CC913) [6]. Another study of the same population found a cluster of strains of SCC*mec*-IV/*spa* t002/PVL+ and SCC*mec*-IV/*spa* t991/PVL−, originating mostly from the community [11].

All these data suggest that SCC*mec* types IV and V are common in hospitals and the community, but the local clonal epidemiology may be significantly different between specific hospitals and geographic locations. In this study, we aimed to describe the molecular epidemiology of MRSA clinical isolates isolated in our facility between 2012 and 2019.

## 2. Methods

### 2.1. Setting

Sanz medical center is a regional, 400-bed hospital, located in the city of Netanya, in central Israel. The hospital admits ∼44,000 patients a year from the community and from ∼14 nearby LTCFs. One LTCF is located within the boundaries of the hospital and is referred to as the local-LTCF (L-LTCF). A policy of “anterior nares screening” was commenced in this facility in 2011, targeting high-risk populations, *i.e.*, patients admitted from LTCFs, patients transferred from another healthcare facility, and patients that were hospitalized during the preceding 1 year before admission. If positive on screening, contact isolation measures were implemented and decolonization attempts were made using chlorhexidine baths and nasal mupirocin 2% for 5 days.

### 2.2. Study Design

We conducted a retrospective study of clinical isolates of *S. aureus* that were cultivated from hospitalized adults (>18 years of age) in our facility between January 2012 and July 2019 and had been *spa*-typed. All MRSA isolates that were found in the bloodstream were routinely sent to the national central laboratories for *spa* typing. Non-bloodstream infection (BSI) MRSA isolates and MSSA isolates were rarely sent for typing. Several MRSA isolates from screening hospitalized patients during an outbreak investigation that occurred in July 2019 were also *spa*-typed. In the MOH central laboratories, all isolates are analyzed by real-time polymerase chain reaction (RT-PCR) for the presence of the *mec*A, *mecC*, and lukS/F-PV genes (Panton Valentine Leukocidin, PVL). *spa* typing analysis is performed for all MRSA and *pvl-*positive MSSA isolates.

*Definitions*. Clinical MRSA isolates, as well as those identified by nasal screening during the first 72 hours from admission, are considered CA, unless the patient had a history of hospitalization during the preceding 1 year or was a LTCF resident. Isolates identified after the first 72 hours were regarded as hospital acquired. Acquisition was defined as either CA or HcA (further divided into hospital acquired and LTCF acquired). Dialysis patients are considered as HcA. The MRSA attribution to a certain ward/facility was determined according to the CDC/NHSN Identifying Healthcare-associated Infections (HAI) surveillance guide [12]. In cases in which MRSA colonization was identified by screening upon admission, and BSI or other clinical isolation of MRSA occurred after 72 hours, the acquisition was attributed according to the screening isolate.

MRSA was defined when the isolate was PCR positive for *mec*A/C gene. Previous carriage state of either MSSA or MRSA was determined according to the hospital laboratory database and medical records.

### 2.3. Microbiology Methods

*S. aureus* isolates were cultured on CNA Columbia Agar and Chromagar *Staphylococcus aureus* media by Hylabs®. MRSA isolates were validated by morphological and biochemical analyses according to the current Clinical and Laboratory Standards Institute (CLSI) and then identified by VITEK 2 system (bioMérieux, France). Screening for oxacillin resistance and for other antimicrobials resistance phenotypes was performed using the KirbyBauer methods or E-test. MRSA strains were sent to the Ministry of Health National Reference Laboratories. There, strains were verified as *S. aureus* and further tested for the presence of *mecA*, *mecC*, and *pvl* by PCR as described previously [11, 13] or by multiplex real-time PCR [14, 15]. *spa* typing was performed as previously described [11] by using the universal primers 1514R and 1113F, and *spa* typing analysis was done using the BioNumerics software. Several *spa*-type representative strains were also typed, by using the method described by Zhang et al. [16], into the major SCC*mec* groups.

This study was approved by the institutional ethics committee of Sanz Medical Center (0102-19-LND).

### 2.4. Statistical Analyses

The following variables were gathered from the medical records of demographics (age, sex, and residency), previous hospitalization dates, previous laboratory data, and death during the index hospitalization, as well as gathered subsequently. Continuous variables were assessed using Student's *t*-test and categorical variables using Chi square or Fisher exact tests. We compared HcA-MRSA with CA-MRSA groups, using the aforementioned tests. We also compared MSSA and MRSA groups, and the percentages of BSI-onset location between HcA- and CA-MRSA groups. All calculations were performed using GraphPad Prism® 7.0 for Windows. Statistical significance was defined when *p* < 0.05.

## 3. Results

### 3.1. *S. aureus* Isolates

**Figure 1:**
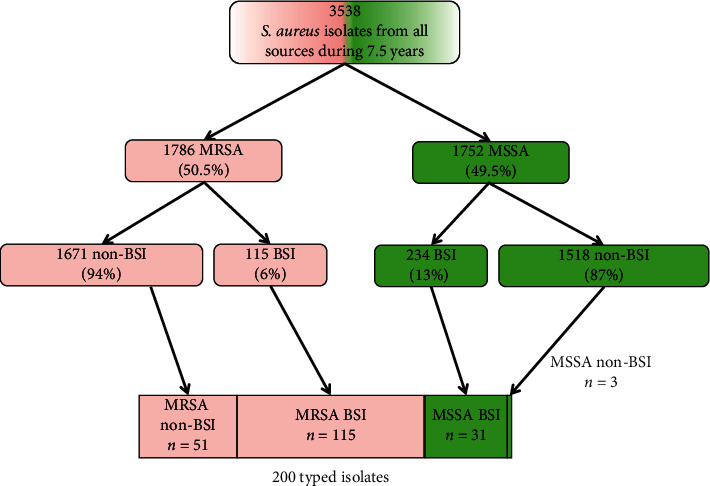
Composition of the 200 *spa*-typed *S. aureus* isolates.

Altogether, during a period of 7.5 years, we had 3538 *S. aureus* isolates identified from all sources, 1786 of which were MRSA (50.5%). Two hundred isolates underwent *spa* typing, and most (166, 83%) were MRSA isolates (including all 115 of the bloodstream MRSA isolates that were found during this period) (Figure 1). The 166 MRSA isolates were obtained from 151 patients: 138 patients had 1 isolate, 11 patients had 2 isolates, and 2 patients had 3 isolates. In contrast with the typing of all MRSA-BSI isolates, only 51 (3%) of non-BSI isolates were typed. These isolates were randomly sent and were obtained from wounds (*n* = 30), abscesses (*n* = 8), pleural fluid (*n* = 2), central catheter tip (*n* = 2), and 9 from nasal screening that were taken as part of an outbreak investigation.

Of 166 MRSA isolates, 145 (87%) were HcA: 80 (48%) were attributed to LTCFs and 65 (39%) to our hospital. The remaining 21 (13%) were CA (Table 1).

Thirty-four MSSA isolates were also typed: 31 (91%) from blood and 3 from sputum, wound, and nasal screening (one each). 23/34 (67%) were CA, 7 were acquired in the hospital, and 4 were acquired in LTCFs.

### 3.2. MRSA *spa* Types

**Figure 2:**
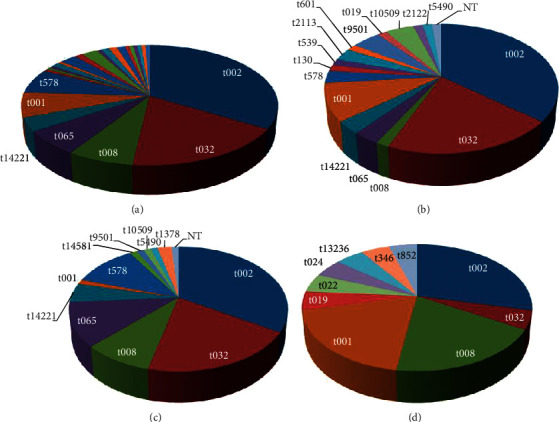
Common MRSA *spa* types according to acquisition location. (a) All (*n* = 166), (b) hospital acquired (*n* = 65), (c) LTCF acquired (*n* = 80), and (d) CA (*n* = 21).

The 166 MRSA isolates belonged to 24 different *spa* types. 153 (92%) were part of a cluster *spa* type (*i.e.*, included more than 1 isolate). The common *spa* type clusters, in descending order, were t002, t032, t008, t001, t065, t578, t14221, t9501, t10509, t2113, t5490, t019, t1378, and t14581 (Table 1, Figure 2. Most isolates (120/166 (72%)) belonged to one of the first 5 types. This aggregation pattern of a relation to a cluster was significantly more prominent among HcA isolates, when compared with CA isolates: 137/145 (94%) and 16/21 (76%), respectively, *p*=0.01.

Of the 145 HcA-related MRSA isolates, the most common *spa* types were t002 (50 isolates, 34%), followed by t032, t008, t065, t578, and t001. Sixty-eight isolates (47%) in this group were typed as SCC*mec*-IV: 48 (33%) were *spa* types t032, t008, t065, and 20 (14%) of other rarer types (t1378, t14221, t578, 1 nontypeable). Only 7 isolates (5%) were SCC*mec*-I (all t001 and 1 nontypeable isolate).

Of the 21 CA-related MRSA isolates, only 5 (25%) were *spa* types that were related to common SCC*mec*-IV: four t008, one t032, and 2 related to rarer types (t13236 and t852). t032 (SCC*mec*-IV) was acquired in the community in only one case, while it was the second most common one among the HcA-MRSA isolates.

Several *spa* type clusters were exclusively HcA. These included t065 (SCC*mec*-IV) and other unique *spa* types (t578, t14211, t9501, t10509, t2113, and t5490).

### 3.3. HcA-MRSA Allocated to LTCFs

The most common types (t002, t032, and t065) were found in most facilities, but several types were unique to specific facilities, such as t578 and t14221 acquired only in the L-LTCF. The epidemic clone t001, which is a commonly reported HcA-MRSA in Israel, was rarely attributed to LTCFs (only 1 isolate during over 7 years). Classical CA-MRSA types, such as t032 (SCC*mec*-IV), t065 (SCC*mec*-IV), and t008 (SCC*mec*-IV), were commonly acquired in LTCFs. t032 was the second most prevalent of the HcA-LTCFs-MRSA strains, after t002.

### 3.4. HcA-MRSA Acquisition in the Hospital

Sixty-five MRSA isolates were attributed to the hospital. The most common *spa* types were t002 (*n* = 23), t032 (*t* = 12), and t001 (*n* = 6). No specific “*spa* type” clusters were found.

### 3.5. CA-MRSA vs HcA-MRSA

There were 132 patients with HcA-MRSA and 19 patients with CA-MRSA. Patients with CA-MRSA infections were younger than HcA-MRSA patients (mean age 67.8 ± 4.4 vs 75.6 ± 1.3 years, respectively, with nearly significant difference, *p*=0.051). Mortality rate due to MRSA-BSI during the index hospitalization was 48%, significantly higher than non-BSI cases (22%), *p*=0.0018. There were no significant differences in mortality rates between the different *spa* types. Mortality rates were similar between CA and HcA patients (32% vs 43%, *p*=0.45). Mortality of patients with BSI from MSSA was significantly lower than BSI from MRSA (7/13, 22.5% vs 55/115, 48%, *p*=0.013).

### 3.6. Bloodstream Infection Acquisition Setting

We studied 115 MRSA-related BSIs. In most cases (88, 77%), BSI onset occurred in the healthcare setting: 64 (56%) in LTCFs and 24 (21%) in the hospital. In 27 patients (23%) BSI occurred in the community setting. In most cases of HcA-BSI (77/88, 87%), the bacteremia occurred in the same facility, in which the MRSA infection was acquired: 55/64 (85%) in the LTCFs and 22/24 (92%) in our hospital. 13/27 (48%) cases, in which MRSA-BSI appeared in the community, were supposedly community acquired since these patients had no previous known exposure to the healthcare system. In the remaining 14 cases, MRSA was suspected to be previously acquired in our hospital (11 cases) or in a LTCF (3 cases). BSI occurrence in the same location of suspected MRSA acquisition was significantly higher in the HcA group as compared with the CA group (87% vs 48%, relative risk = 1.81, confidence interval = 1.3–2.8, *p* < 0.0001).

Thirty-one cases of MSSA-BSI were also studied, 23 of which (74%) developed the bacteremia in the community, and in most cases (21/23, 91%), the infection was also acquired in the community.

### 3.7. HcA-MRSA vs CA-MRSA Antibiograms

The antibiogram profile of the common *spa* clusters is summarized in Table 2, divided into classical HcA-MRSA and CA-MRSA strains. The antibiogram was highly uniform within each *spa* type and also within the groups of HcA-MRSA and CA-MRSA, with the exception of t14221, which was highly resistant to trimethoprim/sulfamethoxazole, and t578, which was resistant to mupirocin. Ciprofloxacin resistance was highly prevalent (95%) in both HcA-MRSA and CA-MRSA. Vancomycin and rifampicin sensitivity were universal in both groups. Sensitivities to erythromycin and to clindamycin were correlated, being significantly different between HcA- and CA-MRSA strains (9% vs 88% and 12% vs 90%, respectively, *p* < 0.0001 in both comparisons). Apart from one *spa* type (t14221), trimethoprim/sulfamethoxazole activity against HcA- and CA-MRSA isolates was excellent.

### 3.8. SCC*mec* Analysis

Of 166 MRSA isolates, 53 *spa* type representative isolates underwent SCC*mec* analysis. There was a high correlation between the *spa* typing and expected SCC*mec* typing in all cases, and the only exception was *spa* type t002, where, of 11 isolates, 7 were SCC*mec*-II, 3 were SCC*mec*-IV, and 1 was SCC*mec*-V.

### 3.9. Specific MRSA *spa* Types

Unique MSRA *spa* types seen in this study include t578 (SCC*mec*-IV) that caused an outbreak in our L-LTFC that involved 11 clinical isolates (7 BSI), and another 7 nasal screening isolates taken from healthcare workers from this ward [17]. This strain has been sparsely reported in the literature, mainly from Germany [18–20], Canada [21], New Zealand [22], and Ireland [23, 24], and is associated with CC22.

*spa* t1378 (SCC*mec*-IV) was found in two screening isolates also from our L-LTCF patients. This is also a rare isolate reported previously from Malaysia [25], Germany [18], United Kingdom [26], and Portugal [27], and was found to be ST22.

*spa* t5490 was found in two isolates (non-BSI) that were acquired in our hospital and a LTCF. This *spa* type was previously reported from Taiwan and was typed SCC*mec*-II [28], but not yet reported from Israel.

*spa* t2113 was found in two clinical isolates (one BSI) in our study, from the same internal ward. We did not have the SCC*mec* typing for these isolates. The t2113 was reported previously with association to CC22, from Germany [20] and from Canada [21].

*spa* t13236 was found once in this study as a BSI-related isolate acquired in the community. To the best of our knowledge, this rare type was previously reported only once, also from Israel [7].

### 3.10. MSSA *spa* Types

Of 34 isolates of MSSA taken from 33 patients, 31 were BSI and 28 were *spa*-typed. Their *spa* types were much more diverse as compared with MRSA strains, with only 2 isolates (7%) typed *spa* t002, and 4 other isolates typed t084 and t223. The remaining 22 isolates were unique (although there were 2 isolates from types commonly reported previously by the central laboratories–t084 and t3454 [4]). Most of the isolates (23/34, 68%) were acquired in the community.

The average age of patients with MSSA was not different from those with MRSA (72.2 vs 74.2, *p*=0.5). Twelve isolates were PVL positive, 6/166 MRSA (3.6%), and 6/34 MSSA (17.6%), *p*=0.0068.

## 4. Discussion

In this hospital-based study, spanning over a period of >7 years, we analyzed 200 isolates of *S. aureus* and correlated their *spa* types to their place of acquisition. Most of the analysis focused on MRSA strains, and the overall clonal data were in accordance with previous reports: the major MRSA *spa* types in our study were t002, t032, and t008, similar to reports from other countries in Europe [3]; to the Israeli Ministry of Health reports of 2016–2017 [4, 5]; and to a recent large community-based survey from Israel [7]. Most (87%) of the MRSA isolates were HcA, and only 21 cases were CA. *spa* type t002 was most commonly encountered, causing 56 (34%) of all MRSA infections. This type is commonly regarded as HcA-MRSA, usually bearing SCC*mec*-II (although frequently also SCC*mec*-IV and V), and indeed 89% of this *spa* type was HcA by our definitions. In contrast, MSSA strains were mostly (67%) CA and showed much higher type diversity. Type clustering was significantly more common in HcA isolates when compared with CA isolates, which probably represents clonal outbreaks and cross-infections among residents of LTCFs and while hospitalized.

As was seen in other studies from Israel, we show that SCC*mec*-IV (classical CA-MRSA related *spa*-types, such as t032, t008) has a major contribution to the overall MRSA burden. Based purely on the antibiogram and previous reports on the correlation between *spa* types and SCC*mec* types [3, 29], it seems that SCC*mec*-IV types constitute at least a half of all strains that are identified in our hospital. If we consider t002 to be SCC*mec*-II, we had 69 antimicrobial phenotypic HcA-MRSA *spa* types (t002, t001, t10509, and t9501) and 67 CA-MRSA types (t032, t008, t065, and others). The same proportions were reported by another hospital-based study from Israel [9]. Also, in concordance with the existing literature, the dichotomy between the acquisition locations of healthcare and community types is undermined, as 40% (67/166) of the healthcare acquired MRSA isolates were in fact CA-MRSA types, while classical CA-MRSA types (such as t032, t008, and t065) were rarely acquired in the community. Among the 21 CA strains, only 5 (24%) were classical community strains (4 of the 5 were t008 and one was t032), and the rest were either t002 or unique strains. t065/SCC*mec*-IV, a CA-MRSA type, which was reported previously from hospitals in Israel [8], was seen in our cohort *only* as a HcA infection, mostly acquired in LTCFs. The European t032/SCC*mec*-IV (EMRSA 15), another classical community strain, was also almost exclusively acquired in LTCFs and in the hospital, as only 1/30 t032 isolates were CA. A similar molecular picture with t032 (EMRSA 15) was described amongst residents of nursing homes in Germany [20], the United Kingdom [30], and Ireland [31]. The same was also true regarding t008/SCC*mec*-IV as 66% (8/12 isolates) of this type were HcA.

Most of the CA isolates (15/21, 71%) and of the HcA isolates (105/145, 72%) belonged to one of the commonly known clusters reported in the past (t002, t032, t008, t001, and t065). Several cluster strains are reported from Israel for the first time, such as *spa* types t578, t14221, t10509, and t9501, as well as others. Types t578 and t14221 were important in our cohort since they were responsible for 37% of the isolates causing outbreaks (of mainly BSIs) in our L-LTCF. Both were typed as SCC*mec*-IV, were clindamycin- and erythromycin-sensitive, and were not previously reported in the literature as causing outbreaks.

Most MRSA infections were attributed to the hospital wards or to LTCFs. *spa* typing analysis of hospital-acquired cases was not helpful in determining a common source or specific type, probably because of the diversity of wards within the facility, lack of typing of screening and of non-BSI clinical isolates, and the presence of multitude of *spa* types. Investigating several common LTCFs showed some predilection for several *spa* types, but the data from these facilities were also partial, since patients are not exclusively transferred to our hospital when infected. Studying the L-LTCF *spa* types over time revealed previously unrecognized clonal outbreaks of specific and unique clones of MRSA (t578 and t14221) [17].

Mortality from MRSA-BSI was high, reaching nearly half of the patients during their index hospitalization, irrelevant of the specific *spa* type or location of acquisition (HcA or CA), even though patients with CA-related MRSA-BSI were slightly younger than HcA patients. MRSA-BSI onset typically (in 77% of cases) occurred in the HcA setting, and usually in the same facility/ward as where the MRSA was acquired, while community onset MRSA-BSI occurred less frequently, and bacterial acquisition was less commonly attributed to the community (11%, 13/115). This means that patients with MRSA-BSI acquired in the community were typically infected previously in the hospital or in LTCFs. This is in contrast with MSSA strains, which usually represent unique community types. MSSA-related bacteremia episodes that occurred in the community conveyed a better prognosis.

This study is limited by being a small-scale, single center study, although it is one of the largest clinical molecular MRSA studies to date from Israel. Another limitation may be our definitions of hospital-acquired infection (HAI). It may be difficult to know the exact location of acquisition of resistant bacteria without performing screening and typing of each isolate upon hospitalization (and even if screening is preformed, false negative results may be an issue). We had to rely on the NHSN surveillance guide to attribute the location of HAIs in this study. The 80 patients that had MRSA infection, identified upon admission to our facility from a LTCF, had an exceedingly high probability that this infection was indeed acquired in their facility. We are also confident that the 21 patients with no previous exposure to the healthcare system had a CA infection. The 65 patients for whom the infection was attributed to our hospital are harder to assign. Forty-seven (72%) of these patients had an infection that was acquired after more than 3 days within the hospital, although it is possible that some were carriers of MRSA on admission; according to the NHSN definitions, they should be regarded as HAIs. The remaining 28% had MRSA infection during the first 3 days of admission, but they were hospitalized in our facility within the preceding year, and we chose to attribute their infection to the hospital. To strengthen the correctness of this attribution, these 18 patients had a similar profile of *spa* typing (with t002, t032, and t001 predominance) as were the other 47 patients in this group.

To conclude, our retrospective *spa* type analysis of BSI-related MRSA strains has confirmed previous results from Israel, showing the predominance of several common European *spa* types and the importance of CA-MRSA strains in the healthcare system. MRSA-related BSI occurred mainly in the HcA setting, from isolates that were acquired in the healthcare system, while CA-related MRSA-BSI is still uncommon.

## Figures and Tables

**Table 1:** Patientsʼ characteristics and *S. aureus* according to *spa* types and allocation of acquisition.

	*spa* type	Isolates (*n*)	Patients (*n*)	Source (BSI/other)	Age, years mean (range)	Males (%)	Acquisition allocated to:			
							Community (%)	Healthcare (%)		
								Total HcA (%)	LTCFs (%)	Hospital (%)
**MRSA**	**MRSA-total**	**166**	**151**	**115/51**	**74.6 (25–99)**	**85 (56)**	**21 (13)**	**145 (87)**	**80 (55)**	**65 (45)**
	t002	56	55	43/13	75.8 (35–94)	28 (51)	6 (30)	50 (34)	27 (33)	23 (35)
	t032	30	27	17/13	73.1 (25–94)	17 (20)	1 (5)	29 (20)	16 (20)	13 (20)
	t008	12	12	10/2	66.5 (35–91)	7 (58)	4 (18)	8 (5)	7 (9)	1 (1)
	t001	11	9	7/4	76.5 (54–97)	6 (54)	4 (18)	7 (5)	1 (1)	6 (9)
	t065	11	11	10/1	87.3 (69–93)	4 (36)	0	11 (8)	9 (11)	2 (3)
	t578	11	10	7/4	76.9 (59–88)	5 (45)	0	11 (8)	9 (11)	2 (3)
	t14221	6	5	6/0	57 (26–81)	3 (60)	0	6 (4)	4 (5)	2 (3)
	t9501	4	4	2/2	65.7 (39–81)	2 (50)	0	4 (3)	1 (1)	3 (5)
	t10509	4	4	1/3	78.5 (51–89)	0	0	4 (3)	1 (1)	3 (5)
	T019	2	2	1/1	49.5 (45, 54))	2 (100)	1 (5)	1 (0.6)	0	1 (1)
	T2113	2	2	1/1	71.5 (70, 73)	2 (100)	0	2 (1)	0	2 (3)
	T1378	2	2	0/2	77 (70, 84)	2 (100)	0	2 (1)	2 (2)	0
	T5490	2	2	0/2	75.5 (69, 82)	2 (100)	0	2 (1)	1 (1)	1 (1)
	Other types^*∗*^	13	13	10/3	75.2 (41–99)	9 (69)	5 (25)	8 (5)	2 (2)	6 (9)

**MSSA**	**MSSA-total**	**34**	**33**	**31/3**	**72.6 (30–103)**	**20 (61)**	**23 (68)**	**11 (32)**	**4 (36)**	**7 (64)**
	t002	2	2	2/0	61.5 (49, 74)	2 (100)	2 (9)	0	0	0
	t223	2	2	2/0	81.5 (71, 92)	2 (100)	2 (9)	0	0	0
	t084	2	2	2/0	92 (81, 103)	0	0	2 (20)	1 (25)	1 (14)
	Other types^*∗∗*^	28	20	25/3	68.4(30–89)	16 (80)	19 (68)	9 (32)	3 (75)	6 (86)

*spa*: *Staphylococcus aureus* protein A; BSI: bloodstream infection; HcA: healthcare associated; LTCF: long-term care facility; L-LTCF: local LTCF; MRSA: methicillin-resistant *Staphylococcus aureus*; MSSA: methicillin-sensitive *Staphylococcus aureus*. ^*∗*^*spa* types: t022, t024, t130, t13236, t14581, t2122, t346, t379, t539, t601, and t852—each had one isolate, 2 isolates were nontypeable. ^*∗∗*^*spa* types: t015, t026, t050, t10275, t12406, t12416, t13445, t1366, t1376, t14581, t15330, t16047, t1614, t1741, t19028, t2612, t267, t306, t3454, t7234, t748, and t786—each had one isolate, 6 were nontypeable.

**Table 2:** Number of isolates and antimicrobials sensitivity of major HcA- and CA-MRSA strains, according to *spa* types^*∗*^.

	*N*	Trimethoprim/sulfamethoxazole	Vancomycin	Ciprofloxacin	Erythromycin	Clindamycin	Rifampicin	Daptomycin	Mupirocin
Classical HcA-MRSA strains									
t002	52	96 (48/50)	100 (49/49)	4 (2/45)	12 (6/51)	15 (8/52)	100 (47/47)	100 (13/13)	62 (8/13)
t001	11	100 (11/11)	100 (11/11)	10 (1/10)	9 (1/11)	9 (1/11)	100 (11/11)	−(0)	100 (2/2)
t10509	4	100 (4/4)	100 (4/4)	0 (0/4)	0 (0/4)	0 (0/4)	100 (4/4)	−(0)	−(0)
t9501	4	100 (4/4)	100 (4/4)	0 (0/4)	0 (0/4)	0 (0/4)	100 (4/4)	−(0)	100 (1/1)
Total	73	97 (67/69)	100 (68/68)	5 (3/63)	10 (7/70)	13 (9/71)	100 (66/66)	100 (13/13)	69 (11/16)
Classical CA-MRSA strains									
t032	29	100 (29/29)	100 (28/28)	8 (2/26)	86 (25/29)	83 (24/29)	100 (28/28)	100 (1/1)	100 (3/3)
t008	12	100 (12/12)	100 (12/12)	10 (1/10)	75 (9/12)	92 (11/12)	100 (12/12)	100 (2/2)	100 (2/2)
t065	11	100 (11/11)	100 (11/11)	0 (0/11)	100 (11/11)	100 (11/11)	100 (11/11)	100 (8/8)	100 (7/7)
t578	9	100 (9/9)	100 (9/9)	0 (0/9)	100 (9/9)	100 (9/9)	100 (9/9)	100 (6/6)	12 (1/8)
t14221	6	16 (1/6)	100 (6/6)	0 (0/6)	100 (6/6)	100 (6/6)	100 (6/6)	−(0)	−(0)
t2113	2	100 (2/2)	100 (2/2)	0 (0/2)	100 (2/2)	100 (2/2)	100 (2/2)	−(0)	−(0)
t5490	2	100 (2/2)	100 (2/2)	0 (0/1)	50 (1/2)	50 (1/2)	100 (2/2)	−(0)	−(0)
Total	71	93 (66/71)	100 (70/70)	5 (3/65)	89 (63/71)	90 (64/71)	100 (70/70)	100 (17/17)	65 (13/20)

^*∗*^Percentages of sensitive strains are presented. Parentheses include the number of sensitive isolates/number of isolates tested.

## Data Availability

The epidemiological and molecular data used to support the findings of this study are available from the corresponding author upon request.
